# An event-related potential comparison of facial expression processing between cartoon and real faces

**DOI:** 10.1371/journal.pone.0198868

**Published:** 2019-01-10

**Authors:** Jiayin Zhao, Qi Meng, Licong An, Yifang Wang

**Affiliations:** Beijing Key Laboratory of Learning and Cognition, Department of Psychology, Capital Normal University, Beijing, China; Temple University, UNITED STATES

## Abstract

Faces play important roles in the social lives of humans. Besides real faces, people also encounter numerous cartoon faces in daily life which convey basic emotional states through facial expressions. Using event-related potentials (ERPs), we conducted a facial expression recognition experiment with 17 university students to compare the processing of cartoon faces with that of real faces. This study used face type (real vs. cartoon), emotion valence (happy vs. angry) and participant gender (male vs. female) as independent variables. Reaction time, recognition accuracy, and the amplitudes and latencies of emotion processing-related ERP components such as N170, VPP (vertex positive potential), and LPP (late positive potential) were used as dependent variables. The ERP results revealed that cartoon faces caused larger N170 and VPP amplitudes as well as a briefer N170 latency than did real faces; that real faces induced larger LPP amplitudes than did cartoon faces. In addition, the results showed a significant difference in the brain regions as reflected in a right hemispheric advantage. The behavioral results showed that the reaction times for happy faces were shorter than those for angry faces; that females showed a higher accuracy than did males; and that males showed a higher recognition accuracy for angry faces than happy faces. Due to the sample size, these results may suggestively but not rigorously demonstrate differences in facial expression recognition and neurological processing between cartoon faces and real faces. Cartoon faces showed a higher processing intensity and speed than real faces during the early processing stage. However, more attentional resources were allocated for real faces during the late processing stage.

## Introduction

Faces play important roles in human social life. They convey unique identity information and basic emotions through facial expressions. In daily life, facial expressions provide important non-verbal forms of information and communication [1]. The ability to recognize a facial expression reflects an individual's ability to infer the psychological states of others [2]. Facial expression recognition not only helps to determine internal emotional states and the intentions conveyed by an individual but also provides feedback and induces social interactions [3,4]. Ekman and Friesen (1978) summarized six basic human facial expressions including happiness, sadness, surprise, fear, anger, and disgust [5]. These facial expressions have been identified and confirmed across different cultural contexts [6].

In addition to real faces, people also encounter many cartoon faces on daily life. Common social networks (e.g., WeChat) provide various cartoon face emoji for communicating and expressing emotions. Compared with real faces, cartoon faces usually have larger eyes, smaller noses, and finer skin texture [7]. Chen and colleagues (2010) found that people developed a preference for real faces with larger eyes after adaption to cartoon faces with unusually large eyes in Japanese cartoons [8]. Some researchers compared cartoon faces and real faces with regard to recognition accuracy and reaction time. Kendall, Raffaelli, Kingstone, and Todd (2016) asked participants to identify emotions on five sets of briefly presented faces that ranged from photorealistic to fully iconic. The results showed stronger emotion recognition accuracy for cartoonized faces [9]. In another study, participants showed faster reaction times to real faces than cartoon faces when they were required to determine whether an image was a face or a car [10]. Moreover, it was specific to real faces instead of cartoon faces that children were more accurate at recognizing upright faces than inverted faces [11]. Similarly, brain imaging studies have also found that the fusiform face area differs between the processing of cartoon faces and real faces [12, 13].

However, research on the recognition of cartoon and real faces has shown mixed results. Using synthesized emotion images, Hoptman and Levy (1988) studied the processing preference of left- and right-handed individuals for cartoon and real faces. The results failed to reveal significant difference between cartoon and real faces [14]. In the comparative study of facial expression processing between Asperger Syndrome children and normal children, no difference was found in processing between cartoon faces and real face [15]. Moreover, Rosset et al. (2008) found that children relied on a configural strategy with all faces when processing emotional expressions of real faces, human cartoon and non-human cartoon faces [16].

In existing researches, cartoon face has no uniform form and definition. In present study, the definition of a cartoon face is a cartoonized face transformed by software called MYOTee (a cartoon image editor, Shenzhen MYOTee Technology Co., Ltd.) according to the characteristics of the real face. It is stylized by a more exaggerated expression and larger eyes, a smaller nose and a more delicate skin than a real face.

Both cartoon and real faces convey emotional information through facial expression. The six basic facial expressions can be categorized as positive or negative expressions. Mixed results have been reported by researches on the reaction times and recognition accuracies of positive and negative facial expressions. Some believe that reaction times for positive expressions are faster than those for other facial expressions [17, 18]. However, there are other studies suggesting that people recognize negative facial expressions faster than positive ones [19, 20].

Event-related potentials (ERPs) were also used to study the neurophysiological basis behind these differences. ERPs can be used to classify different visual stimulus and differentiate disparate emotional states. Without any participant response, ERP testing enables the measurement of emotional attitudes that people are unwilling to express [21].

The ERP components related to faces and facial expressions include N170, VPP, LPP, and others. N170 is primarily distributed in the occipito-temporal region of the brain and usually shows a larger response in the right hemisphere [22]. N170 is a face-specific ERP component, and its peak shows face selectivity. N170 is only induced by face stimuli (i.e., not by furniture, cars, hand gestures, or other stimuli) [23]. Related to face type, research has shown that the N170 component induced by real faces and cars was stronger than cartoon faces and cars [10]. Furthermore, the attractiveness of cartoon faces will affect the amplitude of N170 [24,25]. Another study showed that real faces induced a stronger N170 effect than abstract sketches of faces. Compared with schematic faces, however, the difference was not significant [26]. Facial expressions are also related to N170 during early processing showing that emotional faces induced larger N170 amplitudes than did neutral faces [27–29]. Batty and Taylor (2003) recorded the ERPs of participants responding to the six basic facial expressions and neutral expressions. The results showed that positive expressions resulted in shorter N170 latencies than negative expressions and that fear expressions induced significantly larger amplitudes than did other expressions [1].

N170 has a corresponding positive component at the mind-central sites, namely VPP. VPP and N170 have similar functional properties. They are two manifestations of the same brain processes [30]. VPP sometimes shows more sensitivity to facial expression information than N170, and VPP is influenced by facial expressions when N170 is not [31].

Additional processing of emotional face is reflected by the LPP component which originate from the occipital lobe and the posterior parietal cortex, reflecting the cerebral cortex's evaluation of emotional stimuli, stimulus representation in working memory, and processing of decision making [32–34]. Researchers who investigated adults’ ERP processing of real and cartoon faces with neutral expressions found that real faces induce significantly higher average LPP amplitudes than do cartoon faces [10]. Stronger LPP was also found in neutral real faces compared with neutral puppet faces [35]. Schindler et al. (2017) employed six face-stylization levels varying from abstract to realistic and investigated the difference in the processing of real and cartoon faces showing that the LPP amplitude increased as the faces became more realistic [7]. LPP component is also sensitive to various emotional stimuli including faces [36–40]. The findings related to the influence of emotion valence on LPP are not consistent. Although some reports concluded that negative expressions induce smaller amplitudes than do positive expressions [41], others found that negative expressions induce larger LPP components than do positive expressions [42]. In addition, other studies found no significant differences between the processing of positive and negative expressions [43, 44].

Moreover, other studies have investigated the influence of participant gender on the recognition of facial expressions. Hoffmann and colleagues asked participants to identify six basic but subtle facial emotions (50% emotional content). The results showed that women were more accurate than men at recognizing subtle facial displays of anger, disgust, and fear [45]. Wildgruber, Pihan, Ackermann, Erb and Grodd (2002) found no behavioral difference between males and females with regard to differentiating happy from sad sounds. However, higher response amplitudes within the left-hemisphere posterior middle temporal gyrus were found among women compared with men, whereas a larger increase of activation within the right middle frontal gyrus was observed among the latter [46]. Han, Gao, Humphreys and Ge (2008) found significant differences in the behaviors and brain activities between men and women during emotion-related tasks. Women showed faster threat detection times, while men showed stronger posterior parietal activation [47].

In summary, the existing research suggests that the N170, VPP, and LPP are closely related to facial expression processing but consistent conclusions do not exist regarding the comparison of processing methods, speeds, and intensities between cartoon faces and real faces. With respect to facial expression selection, the present study used anger and happiness for comparison [7, 17, 19, 20, 23, 41]. The present study used an ERP methodology to investigate the processing of real and cartoon facial expressions among men and women. We hypothesized that (1) face type (i.e., real and cartoon faces) would influence the amplitudes and latencies of N170 and VPP though the tendency is not clear; (2) the LPP amplitudes of cartoon faces would be smaller than real faces which are more unique by carrying more details; (3) the late component LPP, but not N170 or VPP, would be affected by emotional valance; (4) recognition time would be faster with regarding to a positive emotion (i.e., happiness) than a negative emotion (i.e., anger); and (5) women would recognize facial expressions faster and more accurately than would men.

## Method

### Participants

We recruited 17 participants (11 males, 6 females; average age = 24.18, SD = 2.32) from universities in Beijing. All participants were right-handed, had normal hearing and vision (with or without correction), and no history of hearing, neurological, or psychiatric disorders. Participants were compensated after the experiment. The current research was approved by the Capital Normal University Institutional Review Board, and written informed consent was obtained from each participant.

### Materials

**Fig 1:**
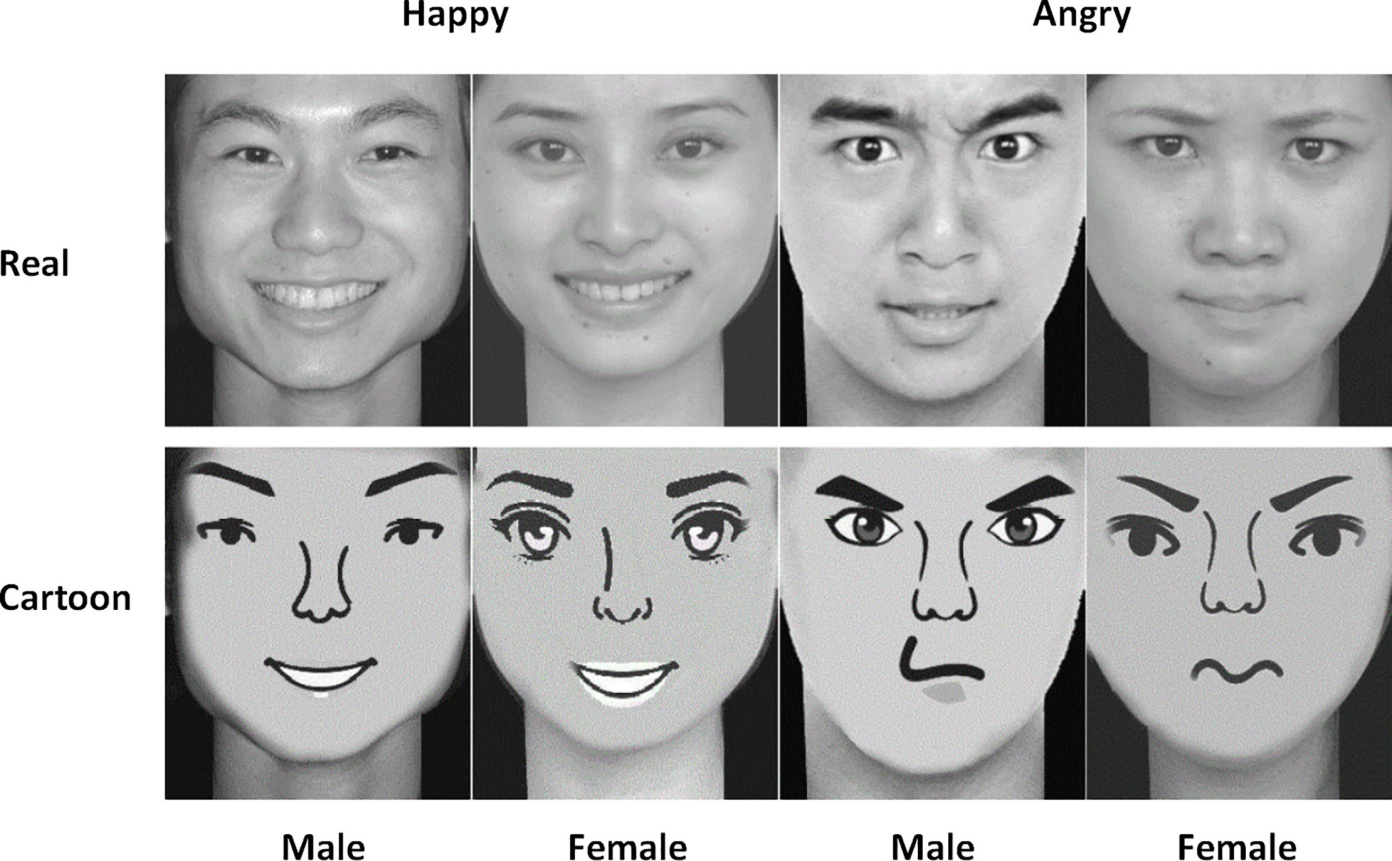
Real and cartoon example faces.

The pictures used in the experiment were selected from the Chinese Facial Affective Picture System (CFAPS; Wang and Luo, 2005) and the Japanese Female Facial Expression (JAFEE) database. Fifty pictures of happy faces (25 males and 25 females) and 50 pictures of angry faces (25 males and 25 females) were selected from the two picture databases. In total, 100 pictures were selected. We used MYOTee (a cartoon image editor) to convert these faces into cartoon faces. Subsequently, we used Photoshop to overlay the cartoon faces onto the original pictures for fine-tuning, and we retained the same face structure and hairstyle to synthesize 100 cartoon facial expression pictures. In total, 200 pictures were used in this experiment. All pictures were presented in black and white with a resolution of 260 × 300 at a consistent contrast (Fig 1). The individuals in Fig 1 have given written informed consent (as outlined in PLOS consent form) to publish these case details.

Twenty additional volunteers (non-participants; mean age = 25.3 years) evaluated the pictures. The evaluation included the identification of facial expression type (i.e., by pressing the “G” key for happiness and the “F” key for anger) and a Likert rating of the facial emotion (9 = extremely happy or angry; 1 = not at all happy or angry). The evaluation results revealed a recognition accuracy of 95.9%, with an emotion intensity rating of 4.81 ± 1.91 (Table 1). Therefore, all 200 pictures were retained as stimuli for the experiment.

**Table 1:** Means (and standard deviations) for accuracy of facial expression recognition and valuation of emotional intensity.

Stimuli Type	Facial Expression	Accuracy(% Correct)	Emotional intensity
**Real**	**Happy**	0.96 (0.21)	4.90(1.85)
**Real**	**Angry**	0.97(0.18)	5.15(2.02)
**Cartoon**	**Happy**	0.97(0.18)	4.34(1.76)
**Cartoon**	**Angry**	0.95(0.23)	4.87(1.94)

### Procedure

**Fig 2:**
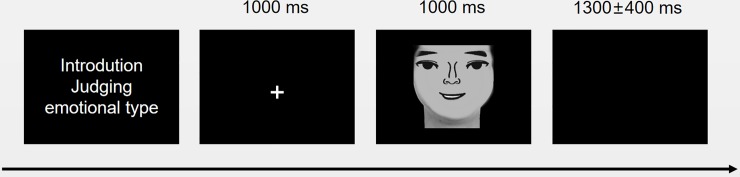
Task timing and example stimulus.

The experiment was conducted in a quiet and dimly lit laboratory. The stimulus images were presented on a 16-inch CRT monitor with a screen resolution of 1920 × 1080. Participants were required to complete facial expression identification tasks according to instructions presented on the monitor. Their electroencephalogram (EEG) data were collected during the experiment. For each trial, a focus point was presented for 1,000 ms. Subsequently, a facial image was presented, and the participant was required to determine whether the face was happy or angry by pressing a button (happy = 1; angry = 2) within 1,000 ms. If a button was pressed within 1,000 ms, then the picture disappeared, and a blank screen was presented until the next picture appeared. If no button was pressed, then the picture disappeared after 1,000 ms, and a blank screen was presented until the next picture appeared. The duration of the blank screens varied randomly from 900 ms to 1,700 ms. Fig 2 shows the experimental procedure. The experiment was divided into two blocks, each with 100 trials. The pictures within each block were balanced. Participants were given 2–3 min to rest between blocks.

### ERP recording and data analyses

The EEG data were collected using an elastic cap from the 64 channel system (HydroCel Geodesic Sensor Net, Electrical Geodesics, Inc., Eugene, OR, USA) with Net Station EEG Software. The impedance of all electrodes was kept below 50 kΩ during data acquisition. All electrodes were physically referenced to Cz (fixed by the EGI system).

Off-line EEG processing and analyses were performed by adopting Net Station EEG Software. The EEG data were band-pass filtered (0.1±40 Hz) and then re-referenced to the average of all electrodes. Trails with artifacts including blink, eye movement and skin potentials where peak to peak deflection exceeding ±80μV and trails with wrong answer were cut off from averaging.

Based on the overall mean chart, the early ERP components (N170 and VPP) generated by the stimuli showed clear peaks. A time window of 125–195 ms was used to measure the ERP peak and peak latency data collected at electrode sites P7 of left hemisphere and P8 of right hemisphere for N170 and electrode site Cz for VPP. For LPP, the average amplitude was calculated with 6 electrode sites (P3, P4, PO3, PO4, Pz, POz) with a time window of 450–650 ms. Latency of LPP component was not included in analysis because that latencies of peaks can vary for components spaning longer time intervals.

For behavioral performances, repeated-measures ANOVAs, with factors gender (male, female), face type (real, cartoon), emotion valence (happy, angry) as independent variables, with RT and accuracy as dependent variables, were conducted. For N170, VPP and LPP components, repeated-measures ANOVAs, with factors gender (male, female), face type (real, cartoon), emotion valence (happy, angry), and lateralization (only for N170) as independent variables, with amplitudes and latency (not for LPP) as dependent variables, were conducted.

## Result

### Behavioral performance

Mean response time (RT) and accuracy and standard deviations are shown in Table 2. For RT, a significant effect of emotion valence, F (1, 15) = 4.95, *p* = 0.042, η_p_^2^ = 0.25, revealed that RT was shorter for happy face than angry face. The main effects of gender and face type did not reach significance, F (1, 15) = 2.40, *p* > 0.05, η_p_^2^ = 0.14, F (1, 15) = 2.17, *p* > 0.05, η_p_^2^ = 0.13, neither did all interactions (*ps* > 0.05).

**Table 2:** Means (and standard deviations) for response time (RT), accuracy.

Condition			RT(ms)	Accuracy(%Correct)
**Real**	**Happy**	**Male**	608.22(72.05)	90.28(6.60)
		**Female**	552.13(76.48)	96.14(2.83)
		**Total**	581.83(77.38)	93.04(5.86)
	**Angry**	**Male**	607.48(70.39)	90.68(6.33)
		**Female**	553.29(75.85)	96.22(2.71)
		**Total**	581.98(75.97)	93.29(5.60)
**Cartoon**	**Happy**	**Male**	606.93(70.10)	90.57(6.21)
		**Female**	550.89(78.29)	96.19(2.72)
		**Total**	580.56(77.27)	93.22(5.56)
	**Angry**	**Male**	608.02(70.46)	90.62(6.12)
		**Female**	553.83(75.63)	96.08(2.92)
		**Total**	582.05(76.10)	93.19(5.51)

For accuracy, a significant effect of gender, F (1, 15) = 5.38, *p* = 0.035, η_p_^2^ = 0.26, indicated that the female performed better than the male. Results also showed a significant interaction between gender and emotion valence, F (1, 15) = 5.50, *p* = 0.033, η_p_^2^ = 0.27. Follow-up simple effect analysis showed that for the male, performance was better in condition of angry face than happy face, F (1, 15) = 10.48, *p* < 0.01, η_p_^2^ = 0.41; for the female, accuracy did not differ for happy face versus angry face, F (1, 15) = 0.03, *p* > 0.05, η_p_^2^ = 0.002. The main effects of face type and emotion valence did not reach significance, F (1, 15) = 0.34, *p* > 0.05, η_p_^2^ = 0.02, F (1, 15) = 4.40, *p* > 0.05, η_p_^2^ = 0.23, neither did other interactions (*ps* > 0.05).

### N170

Mean amplitudes and latency of N170, VPP, LPP and standard deviations are shown in S1 Table. For N170 amplitudes, since the main effect of emotion valence is not significant, F (1, 13) = 1.03, *p* > 0.05, η_p_^2^ = 0.07, we analyze the data separately in two cases. In the cases of both happy faces and angry faces, there were significant effects of face type, F (1, 13) = 28.58, *p* < 0.01, η_p_^2^ = 0.69, F (1, 13) = 34.90, *p* < 0.01, η_p_^2^ = 0.73, revealed that amplitudes were bigger for cartoon face than real face. However, only for happy faces, results showed a significant interaction between face type and lateralization, F (1, 13) = 10.12, *p* < 0.01, η_p_^2^ = 0.44. Follow-up simple effect analysis showed that for right hemisphere, amplitude was bigger in condition of cartoon face than real face, F(1, 13) = 40.71, *p* < 0.01, η_p_^2^ = 0.76; for left hemisphere, amplitude did not differ between cartoon and real face, F (1, 13) = 3.58, *p* > 0.05, η_p_^2^ = 0.22. The main effects of gender and lateralization did not reach significance, F (1, 13) = 0.61, *p* > 0.05, η_p_^2^ = 0.05, F (1, 13) = 2.32, *p* > 0.05, η_p_^2^ = 0.15, neither did other interactions (*ps* > 0.05).

For N170 latency, since the main effect of emotion valence is not significant, F (1, 13) = 0.04, *p* > 0.05, η_p_^2^ = 0.003, we analyze the data separately in two cases. In the cases of both happy faces and angry faces, there were significant effects of face type, F (1, 13) = 5.95, *p* = 0.03, η_p_^2^ = 0.31, F (1, 13) = 4.94, *p* = 0.045, η_p_^2^ = 0.28, indicated that latency was longer for real face than cartoon face. However, only for angry faces, results showed a significant interaction between face type and lateralization, F (1, 13) = 5.68, *p* = 0.033, η_p_^2^ = 0.30. Follow-up simple effect analysis showed that for right hemisphere, latency was longer in condition of real face than cartoon face, F(1, 13) = 26.09, *p* < 0.01, η_p_^2^ = 0.67; for left hemisphere, latency did not differ between real face and cartoon face, F (1, 13) = 0.12, *p* > 0.05, η_p_^2^ = 0.01. The main effects of gender and lateralization did not reach significance, F (1, 13) = 0.69, *p* > 0.05, η_p_^2^ = 0.05, F (1, 13) = 0.04, *p* > 0.05, η_p_^2^ = 0.003, neither did other interactions (*ps* > 0.05).

### VPP

For VPP amplitudes, since the main effect of emotion valence is not significant, F (1, 13) = 3.71, *p* > 0.05, η_p_^2^ = 0.22, we analyze the data separately in two cases. Only in the cases of happy faces, a significant effect of face type, F(1, 13) = 9.54, *p* < 0.01, η_p_^2^ = 0.42, revealed that for happy faces amplitudes were bigger for cartoon face than real face. The main effect of gender did not reach significance, F (1, 13) = 3.21, *p* > 0.05, η_p_^2^ = 0.20, neither did all interactions (*ps*>0.05).

For VPP latency, results showed that interaction between face type and emotion valence was significant, F (1, 13) = 6.80, *p* = 0.022, η_p_^2^ = 0.34. Follow-up simple effect analysis found no significant effect. For real face, latency in condition of happy face was 161.04(3.15) ms, and it was 164.90(2.71) ms in condition of angry face. For cartoon face, latency in condition of happy face was 159.88(3.16) ms, and it was 158.11(3.36) ms in condition of angry face. The main effects of gender, face type and emotion valence did not reach significance, F (1, 13) = 2.15, *p* > 0.05, η_p_^2^ = 0.14, F (1, 13) = 1.50, *p* > 0.05, η_p_^2^ = 0.10, F (1, 13) = 0.64, *p* > 0.05, η_p_^2^ = 0.05, neither did other interactions (*ps* > 0.05).

### LPP

For LPP amplitudes, Since the main effect of emotion valence is not significant, F (1, 13) = 3.09, *p* > 0.05, η_p_^2^ = 0.19, we analyze the data separately in two cases. Only in the cases of angry faces, a significant effect of face type, F(1, 13) = 7.08, *p* = 0.02, η_p_^2^ = 0.35, revealed that for angry faces amplitudes were bigger for real face than cartoon face. The main effect of gender was not significant, F (1, 13) = 3.03, *p* > 0.05, η_p_^2^ = 0.19, neither were all interactions (*ps* > 0.05).

## Discussion

The differences in the processing of the two face types were primarily reflected by the amplitudes and latencies of N170, VPP, and LPP. Cartoon faces were associated with significantly higher amplitudes than real faces on N170 and VPP. And cartoon faces resulted in shorter N170 latencies than did real faces. In addition, for N170 there was a significant interaction between face type and lateralization showing larger amplitudes and shorter latencies for cartoon faces in the right hemisphere. Real and cartoon faces also differed in LPP amplitudes which induced by real faces were significantly larger than those induced by cartoon faces. Although no significant effect of emotion valence and gender was found with regard to ERP components, happy faces resulted in shorter reaction time than did angry faces and the females were more accurate than the males. Moreover, there was a significant interaction between emotion valence and gender showing that the males recognized angry faces more accurately than happy face whereas no difference was found in the females.

For the early components N170 and VPP, cartoon faces induced larger amplitudes and shorter latencies. This finding suggests that cartoon facial expressions are more easily recognized than real facial expressions, that is inconsistent with the results of previous studies that have indicated that real faces induce larger N170 and VPP amplitudes and shorter latencies than do cartoon faces [10, 26]. These inconsistent results might have been caused by the differences in stimulus materials. In Wang’s study, real faces were collected from preschool children (mean age = ~6 years), and cartoon faces were obtained from screenshots of high-resolution popular cartoon DVDs which have various face shapes and facial features[10]. In the present study, the real face stimulus were collected from adult facial expression databases (CFAPS and JAFFE), and the cartoon faces were converted from these real faces which have uniformed face shapes and facial features. Therefore, different facial structures may lead to inconsistent results. Also, The cartoon faces used in this study are more simplified and abstract and, therefore, might have resulted in stronger N170 amplitudes. Schindler et al. (2017) employed six face-stylization levels varying from abstract to realistic and investigated the difference in the processing of real and cartoon faces [7]. The results revealed a U-shape relationship between N170 and face realism. That is, both the most abstract and most realistic faces caused stronger reactions compared with medium-stylized faces. In addition, Proverbio, Riva, Martin and Zani (2010) found that infant faces elicited higher N170 amplitudes than did adult faces, most likely because of juvenile characteristics such as the larger proportion of the eyes [48]. In the present study, the eyes of the cartoon faces were much larger than those of real people.

Real and cartoon faces also differed in LPP amplitude. The LPP amplitudes induced by real faces were significantly larger than those induced by cartoon faces. This finding is consistent with those of previous studies [7, 10, 49]. When the neutral expressions of real faces and puppet faces were compared, no differences in N170 were observed. However, a stronger LPP was found with regard to real faces starting at 400 ms [49]. This effect is probably because of the uniqueness of the real face as well as the understanding of the portrayed individual [35], considering that computer-generated faces are usually more difficult to remember [50, 51]. Bruce and Young (1986) considered facial feature encoding and identify recognition as the second stage of face recognition [52]. This stage includes the accurate processing of facial information such as age, gender, race, and facial expression. Besides, compared with simplified cartoon faces, real faces convey more personal information and social meaning. Thence adults may invest more psychological resources to real faces during late face processing. Furthermore, LPP is related to facial attractiveness [49, 53, 54]. Therefore, the results of the present study might suggest that real faces are more attractive than simplified cartoon faces to adults.

For behavioral data, reaction time was shorter for happy faces than for angry faces, and response accuracy was better for the female than for the male, which were consistent with previous studies. Positive facial expressions are more easily recognized than negative facial expressions [17, 18] and women have a face recognition advantage over men [55–58]. In addition, an interaction effect was found between emotion valence and gender. Men showed a higher accuracy for recognizing angry faces, whereas women showed no difference. A possible explanation is that women are better at identifying emotion in general, and in the conditions of both happy faces and angry faces there maybe is a ceiling effect on accuracy but the two emotions are more difficult for men. The high accuracy of angry face recognition among men might be because they are more physically aggressive than women [59]. Therefore, men are likely more sensitive to social signals that convey aggressiveness.

Our selected stimuli (i.e., the cartoon and real facial expression pictures) represent the advantage of this study. One important advantage is that the cartoon faces were converted from real faces and therefore retain the same facial structure and hairstyle. Most of the existing research has used screenshots of cartoon characters or sketched faces and expression icons [10, 20, 26]; therefore, they cannot exclude nuanced information other than facial expressions compared with real faces. Another advantage is that all of the images used were of Asian adults, which prevented the introduction of cultural and age differences that might have been caused by use of western emotional faces. One limitation of this study is its limited sample size (17 participants), which might have resulted in the large standard deviation in VPP latency. Although the interaction effect of face type and emotion valence was significant, the simple effects were not. However, in addition to the significant main effect of faces type for ERP components, these η_p_^2^ were also considerable (all η_p_^2^ > 0.20). But due to the sample size, there is still such a possibility that these findings are suggestive and may not be rigorous. Future research should increase the sample size to examine the interaction of cartoon and real faces with regard to facial expression type. Another limitation is that although ERP is advantageous for its temporal resolution, its special resolution is low. Future research should apply fMRI, which has a high spatial resolution. Finally, children are the primary audience for cartoons. Childhood is an important stage for developing emotional cognition. Based on the present study, future studies should compare adults and children with regard to the processing of cartoon and real facial expressions. This line of research might help draw a clearer picture of the developmental process associated with cartoon face processing.

## Conclusions

We used ERPs to measure the brain activity responses induced by the facial expressions of cartoon and real faces. According to the neurophysiological evidence in this study, face type has a strong but heterogeneous effect on the N170, VPP, and LPP components. During the early processing stage, adults process cartoon faces faster than real faces. However, adults allocate more attentional resources for real face processing during late processing stage. Future research should use larger sample sizes to examine the interaction between face type (real vs. cartoon) and facial expression.

## Supporting information

S1 Table Means (and standard deviations) for amplitudes and latency of N170, VPP and LPP. (PDF)
