# Supplementary material for: An event-related potential comparison of facial expression processing between cartoon and real faces
Source: PLoS One. 2019 Jan 10;14(1):e0198868. doi: 10.1371/journal.pone.0198868 (PMC6328201; doi:10.1371/journal.pone.0198868)
Supplement: S1 Table — (PDF) [file pone.0198868.s001.pdf]

S1 Table. Means (and standard deviations) for amplitudes and latency of N170, VPP and LPP.

Condition			N170-Amplitude(μV)		N170-Latency(ms)		VPP-Amplitude (μV)	VPP-Latency (ms)	LPP-Amplitude (μV)
			P7	P8	P7	P8			
Happy	Real	Male	-8.22(4.05)	-7.08(5.56)	162.07(13.97)	166.96(6.54)	5.94(2.71)	165.18(7.77)	2.97(2.29)
		Female	-6.37(2.15)	-10.12(2.84)	163.23(9.16)	159.56(14.43)	3.97(1.80)	156.89(16.56)	2.21(3.26)
		Total	-7.48(3.45)	-8.30(4.79)	162.53(11.91)	164.00(10.62)	5.16(2.52)	161.87(12.25)	2.67(2.64)
	Cartoon	Male	-8.43(4.00)	-9.63(6.61)	160.54(15.35)	163.43(7.33)	6.38(2.23)	164.54(9.83)	2.47(3.52)
		Female	-8.34(3.65)	-14.37(2.75)	159.89(8.98)	156.56(11.13)	5.65(1.84)	155.22(14.80)	-1.28(3.39)
		Total	-8.39(3.72)	-11.53(5.78)	160.28(12.79)	160.68(9.33)	6.09(2.05)	160.81(12.48)	0.97(3.85)
Angry	Real	Male	-7.29(4.07)	-7.11(5.51)	163.68(10.99)	167.02(8.38)	5.63(2.75)	166.57(8.58)	3.57(2.28)
		Female	-6.24(3.11)	-9.75(3.83)	158.22(11.87)	161.56(9.36)	2.85(1.40)	163.22(12.52)	2.27(1.11)
		Total	-6.87(3.64)	-8.17(4.94)	161.50(11.27)	164.83(8.89)	4.52(2.64)	165.23(10.05)	3.05(1.96)
	Cartoon	Male	-8.50(5.22)	-9.60(5.96)	161.43(12.59)	162.76(7.15)	6.08(2.42)	162.32(9.57)	2.58(2.55)
		Female	-8.28(3.13)	-12.77(2.68)	161.57(9.18)	157.57(11.14)	4.26(2.82)	153.90(16.60)	1.14(1.16)
		Total	-8.42(4.37)	-10.87(5.05)	161.49(10.99)	160.69(8.97)	5.35(2.65)	158.95(13.00)	2.00(2.17)
